# Growth performance and gut health of broilers fed heat- and enzyme-treated *Vigna unguiculata* and *Cajanus cajan* diets

**DOI:** 10.3389/fphys.2025.1561426

**Published:** 2025-06-10

**Authors:** Filomena Dos Anjos, Julia Dibner, Frances Yan, Mercedes Vazquez-Anon, Ellen. S. Dierenfeld, Abilio P. Changule, Manuel Garcia-Herreros, Custódio G. Bila, Michael Chimonyo

**Affiliations:** ^1^ Eduardo Mondlane University, Faculty of Veterinary Medicine, Maputo, Mozambique; ^2^ Novus International, Inc., Chesterfield, MO, United States; ^3^ Ellen S. Dierenfeld, LLC, StLouis, MO, United States; ^4^ Agricultural Research Institute of Mozambique, Directorate of Animal Science, Matola, Mozambique; ^5^ National Institute for Agricultural and Veterinary Research (INIAV), Santarém, Portugal; ^6^ CIISA-AL4AnimalS, Faculty of Veterinary Medicine, University of Lisbon, Lisbon, Portugal; ^7^ Department of Research and Development, Intermed Mozambique Lda, Maputo, Mozambique; ^8^ Center of Excellence in Agri-Food Systems and Nutrition (CEAFSN) - Eduardo Mondlane University (UEM), Maputo, Mozambique; ^9^ University of Venda, Faculty of Science, Engineering and Agriculture Thohoyandou, Thohoyandou, South Africa

**Keywords:** broiler diets, crypt depth, roasting, phytases, poultry

## Abstract

**Introduction:**

Optimizing broiler production performance while maintaining digestive health is a key challenge in poultry management. Dietary modifications, including the use of alternative protein sources and enzyme supplementation, can influence growth and gut health.

**Methods:**

This study evaluated the effects of roasting, extrusion, and enzymatic supplementation of *Vigna unguiculata* (cowpeas) and *Cajanus cajan* (pigeon peas) on growth performance and gut health in broilers. A total of 210 one-day-old Ross male broilers were randomly allocated to seven dietary treatments (five replicates per treatment; six broilers per pen) in a completely randomized design. Experimental diets incorporated raw or heat-treated cowpeas or pigeon peas, with or without a mixed enzyme supplement (protease, xylanase, and phytase), replacing soybean meal at 400 g/kg inclusion.

**Results:**

Roasting cowpeas or enzyme supplementation did not significantly affect body weight gain or cumulative performance index (p > 0.05). Roasting pigeon peas significantly improved chick performance (p < 0.05). Broilers fed diets containing raw cowpeas, raw cowpeas with enzymes, and both roasted and raw pigeon peas (with or without enzymes) exhibited reduced duodenal crypt depth compared to the control (p < 0.05). Furthermore, raw cowpeas, irrespective of enzyme supplementation, induced an immune response in the duodenum that was not observed in broilers fed the control diet, roasted cowpeas, or raw pigeon peas.

**Discussion:**

Partial substitution of maize and soybean meal with 400 g/kg roasted pigeon peas is a promising alternative in broiler nutrition, enhancing both performance and gut health. However, raw cowpeas may trigger an intestinal immune response, highlighting the importance of processing methods.

## 1 Introduction

Chickens play a crucial role in food security and income generation for rural households, serving as primary sources of meat and eggs (Kumar et al., 2021). However, productivity in smallholder production systems is often constrained by challenges such as high disease prevalence and limited access to high-quality feed (Wilson et al., 2022). Traditional protein sources, such as soybean meal, fishmeal, and bone meal, are commonly used in chicken diets because of their high protein content and critical amino acid profiles, which are necessary for growth and egg production (Nunes et al., 2022). Soybean meal, in particular, is both protein-rich and cost-effective, supporting optimal poultry development (Ghazalah et al., 2022).

Despite these benefits, conventional protein sources present several drawbacks. The increasing price volatility and sustainability concerns surrounding soybean production, the risks of overfishing and inconsistent nutrient composition in fishmeal, and the potential pathogen contamination in improperly processed bone meal highlight the need for alternative feed ingredients (Barra et al., 2021; Nguyen et al., 2021). In this context, exploring locally available protein sources is essential for enhancing poultry nutrition and sustainability.

Cowpeas (*Vigna unguiculata*) and pigeon peas (*Cajanus cajan*) have emerged as promising alternatives, particularly in smallholder systems, because of their affordability and accessibility (Manole et al., 2024). Besides serving as nutrient-dense feed ingredients, cowpeas provide both edible grains and tender leaves for household consumption, while pigeon peas are widely cultivated by resource-limited farmers, contributing to improved nutrition and food security (Manyelo et al., 2022; Oppewal and da Cruz, 2017; Kuraz Abebe, 2022).

While cowpea (*V. unguiculata*) and pigeon pea (*C. cajan*) offer several advantages, including high protein content and cost-reducing potential in poultry diets, their utilization is constrained by nutritional challenges. These legumes contain anti-nutritional factors (ANFs) and high fiber levels, which can hinder nutrient absorption and negatively impact gut health in poultry (Salim et al., 2023; David et al., 2024). Key ANFs, such as trypsin inhibitors, polyphenolic compounds, and phytic acid, reduce digestive efficiency and overall growth performance (Mamo et al., 2018; Samtiya et al., 2020). Moreover, the high fiber content in these plant-based feeds is not readily digestible by chickens, underscoring the potential benefits of exogenous enzyme supplementation to enhance the breakdown of non-starch polysaccharides and improve feed efficiency (Nguyen et al., 2021; Alagawany et al., 2018).

Cowpeas and pigeon peas can account for up to 50% of the diet in smallholder poultry systems (Dos Anjos et al., 2015). Several processing strategies have been developed to reduce the negative effects of ANFs and increase nutrient bioavailability, increasing the nutritional value of these legumes. Common methods include soaking, cooking, roasting, and extrusion. Soaking reduces anti-nutritional compounds while cooking and roasting gelatinized starches, improving nutrient digestibility (Suhag et al., 2021). Extrusion processing, which employs high temperature and pressure, further modifies plant structure, decreasing ANF concentrations and enhancing protein digestibility (Boriga et al., 2023).

Beyond physical processing, enzymatic supplementation has gained prominence in poultry nutrition. Phytase and xylanase are enzymes that hydrolyze phytic acid and soluble fibers, respectively, which improve nutrient absorption (Alagawany et al., 2018). These additives effectively mitigate the negative effects of fiber and ANFs, leading to enhanced growth performance and gut health in broilers (Nguyen et al., 2021). The integration of processing techniques with enzyme supplementation presents a promising strategy for optimizing the incorporation of cowpeas and pigeon peas in poultry diets, contributing to more sustainable and efficient production systems.

This study hypothesizes that roasting cowpeas and pigeon peas will improve gut health and broiler performance compared to diets containing unprocessed legumes. It also assesses the effects of supplemental enzyme mixtures combined with roasted and unroasted legume diets on gut health and broiler production performance.

## 2 Materials and methods

### 2.1 Ethical statement

This study was reviewed and approved by the Ethical Committee on Animal Use Rights and Welfare of the Faculty of Veterinary Medicine, Eduardo Mondlane University (Record No. ECAURW-EMU-10/2024), Maputo, Mozambique.

### 2.2 Reagents and media

Unless stated otherwise, all sample analyses were conducted in-house at the Novus International, Inc. Analytical Laboratory, St. Charles, MO, United States.

### 2.3 Grain legumes

Grains of *Vigna unguiculata* (INIA 36) and *Cajanus cajan* (IT 82–78) cultivars, totaling 1.0 kg, were supplied by the National Research Institute of Agriculture (IIAM). Both legumes were cultivated under uniform agronomic conditions in Maputo, Mozambique.

### 2.4 Determination of grain-derived urease activity

Raw beans were initially ground using a Retsch cutting mill (Haan, Germany) with a 2 cm × 2 cm screen, followed by a second grinding step using a 1 cm × 1 cm screen to achieve a final particle size of 1.5 mm. Roasting was performed in a hotbox oven at 120°C for 45 min, after which the seeds were cooled and ground again through a 1.5 mm sieve.

To assess the reduction of trypsin inhibitors following heat treatment, urease activity was analyzed in duplicate using the method described by the Association of Official Analytical Chemists (1980). A finely ground 0.2 g (±0.001 g) sample was mixed with 10 mL of buffered urea solution in a test tube. Blank samples comprise an identical amount of ground sample combined with 10 mL of phosphate buffer solution. The tubes were capped, gently swirled (without inversion), and placed in a water bath maintained at 30°C, with a 5-min interval between test and blank samples.

The test and blank tubes were swirled every 5 minutes for 30 min of incubation. Following incubation, the tubes were removed from the water bath, briefly cooled, and approximately 5 mL of the supernatant was transferred into a beaker, where a pH meter electrode was immersed in the liquid. The pH of the supernatant was measured approximately 5 minutes after removal from the water bath, and the difference between the pH of the test and blank samples was calculated as an index of urease activity.

### 2.5 Experimental design and experimental birds

The housing and care of the animals throughout the experiment adhered to the Guide for the Care and Use of Agricultural Animals in Research and Teaching (National Research Council, 2011). All research procedures were reviewed and approved by the animal ethics committee, which included members from Novus International Inc. (20 Research Park Drive, St. Charles, MO 63304) and a licensed veterinarian from Bridgeton Animal Hospital (3148 McKelvey Road, Bridgeton, MO 63044). 210 one-day-old male Ross 708 broiler chicks were obtained from a commercial hatchery. Upon arrival, the chicks were weighed, wing-banded for identification, and housed in stainless steel chick batteries for 15 days. Initially, all birds were maintained under identical nutritional and environmental conditions from hatch until day 14. They were provided with a standard diet formulated to meet their nutritional requirements and had *ad libitum* access to water. The chicks had uniform initial body weights (approximately 41 g per bird).

The experiment followed a completely randomized design, with five replicate pens of six chicks assigned to each of seven dietary treatments. House temperature was recorded three times daily throughout the study. During the first week, the temperature was maintained at 35°C, gradually decreasing to 30°C by the end of the experimental period. The lighting regimen was adjusted over time: from days 5–9, chicks were exposed to 24 h of light at an intensity of two candles, while from days 10–14, the schedule was modified to 20 h of light (2 foot-candles) followed by 4 h of darkness. Bird health was checked at least twice daily, with any anomalies or symptoms of distress noted. Mortalities were documented, including the body weights of deceased chicks.

### 2.6 Diets

A maize-soybean meal-based basal diet in mash form was formulated to meet the nutritional requirements of broiler chicks from 1 to 14 days post-hatch, following the recommendations of the National Research Council (National Research Council, 1994). Acid-insoluble ash (AIA, 1%) was an indigestible marker for digestibility measurements. Table 1 presents the ingredient composition of the basal diet and the six dietary treatments used in the study. The first treatment (T1) served as the control diet, comprising maize and soybean meal. Treatments 2 (T2), 3 (T3), and 4 (T4) included 400 g/kg of *Vigna unguiculata*. Specifically, T2 contained raw cowpeas, T3 included roasted cowpeas, and T4 comprised raw cowpeas supplemented with enzymes. Treatment 5 (T5) replaced soybean meal with raw pigeon peas, while T6 included roasted pigeon peas at the same inclusion level (400 g/kg). Treatment 7 (T7) comprised raw pigeon peas with enzyme supplementation.

**TABLE 1:** Dietary treatments used in the study.

Ingredients (g/kg)	T1	T2	T3	T4	T5	T6	T7
Maize	593.4	351.4	351.4	379.3	351.4	379.3	351.4
Soybean	318.1	168.1	168.1	140.9	168.1	140.9	168.1
Cowpeas	0.0	40.0	40.0	40.0	0.0	0.0	0.0
Pigeon peas	0.0	0.0	0.0	0.0	40.0	40.0	40.0
Vegetable oil	10.0	33.3	33.3	10.0	33.3	10.0	33.3
Dicalcium phosphate	18.0	18.6	18.6	18.0	18.6	18.0	18.6
Limestone	11.1	11.4	11.4	11.8	11.4	11.8	11.4
Salt	3.6	3.5	3.5	3.5	3.5	3.5	3.5
NaHCO_3_	2.6	2.8	2.8	2.7	2.8	2.7	2.8
Alimet^©^	1.9	3.2	3.2	3.2	3.2	3.2	3.2
L-Lysine HCl 78%	1.3	1.3	1.3	1.7	1.3	1.7	1.3
Threonine	0.7	1.4	1.4	1.3	1.4	1.3	1.4
Tryptophan	0.0	0.0	0.0	0.0	0.0	0.0	0.0
Choline CL 60%	0.8	1.9	1.9	2.0	1.9	2.0	1.9
Trace mineral mix	2.0	2.0	2.0	2.0	2.0	2.0	2.0
Vitamin mix	0.5	0.5	0. 5	0. 5	0. 5	0. 5	0.5
Mold Guard	0.5	0. 5	0. 5	0. 5	0. 5	0.5	0.5
Santoquin-Mix6	0.1	0. 1	0. 1	0. 1	0.1	0.1	0.1
Sand	35.4	0.0	0.0	22.5	0.0	22.5	0.0
Total	1,000	1,000	1,000	1,000	1,000	1,000	1,000

T1: control; T2: raw cowpea; T3: roasted cowpea; T4: raw cowpea with enzymes; T5: raw pigeon pea; T6: roasted pigeon pea; T7: Raw pigeon pea with enzymes. Ingredients include NaHCO_3_ (sodium bicarbonate); Alimet (Novus International, St. charles, MO, United States; 88% active methionine source); trace mineral mix supplied per kilogram of diet: FeSO_4_·H_2_O, 40 mg; calcium iodate, 1.25 mg; sodium selenite, 0.3 mg; Mintrex® Zn, 64 mg; Mintrex® Cu, 16 mg; Mintrex® Mn, 64 mg (all Mintrex® products sourced from Novus International, St. charles, MO, United States); vitamin mix supplied per kilogram of diet: retinol, 9.2 mg; cholecalciferol, 100 μg; dl-α-tocopherol, 90 mg; menadione, 6 mg; thiamine, 6.2 mg; riboflavin, 26.5 mg; pantothenic acid, 39.7 mg; niacin, 100 mg; pyridoxine, 11 mg; folic acid, 4 mg; biotin, 0.3 mg; cyanocobalamin, 0.1 mg; Mold Guard (a feed preservative for mold control; Kemin, Des Moines, IA, United States); Santoquin Mix 6 (feed preservative; Novus International, St. charles, MO, United States).

The enzyme blend used in the study comprises a commercial phytase (Ronozyme; DSM Nutritional Products Ltd., Basel, Switzerland), a protease (Cibenza DP100; Novus International, St. Charles, MO, USA), and a combination of xylanase, α-galactosidase, and β-glucanase (Cibenza CSM; Novus International, St. Charles, MO, USA). The enzyme premix was prepared by mixing 60 g of each enzyme product with 5.82 kg of ground maize. This enzyme combination was specifically chosen to address the anti-nutritional challenges posed by cowpeas and pigeon peas, which are known to contain phytic acid, protease inhibitors, and non-starch polysaccharides (NSPs). Phytase was included to hydrolyze phytic acid and release bound phosphorus; protease to enhance protein digestion and mitigate the effects of trypsin inhibitors; and the carbohydrases (xylanase, β-glucanase, and α-galactosidase) to reduce digesta viscosity and improve the breakdown of NSPs and indigestible oligosaccharides. The selected enzymes and their inclusion levels were based on current industry recommendations and published literature, which support their efficacy in legume-rich poultry diets (Bedford and Apajalahti, 2022; Nguyen et al., 2021; Alagawany et al., 2018). These enzymes have been shown to improve nutrient availability, enhance gut health, and reduce intestinal fermentation caused by undigested substrates. The blend and dosage were also chosen for their feasibility in smallholder settings, where low-cost and scalable feed solutions are critical. The nutritional composition of each diet is presented in Table 2, while Table 3 provides the average chemical composition of the cowpea and pigeon pea used in the study. The proximate composition of the raw and roasted cowpeas and pigeon peas used in this study are presented in Table 2 while Table 3 provides the average chemical composition and Table 4 Anti-nutritional factors to provide insight into their nutritional profiles and the basis for their dietary inclusion.

**TABLE 2:** Calculated nutritional composition of the diets (as fed basis).

Nutrient composition	Treatments						
	T1	T2	T3	T4	T5	T6	T7
ME (kcal/kg)	2,900	2,900	2,900	2,900	2,900	2,900	2,900
Crude protein (g/kg)	204	196	196	201	196	201	196
Available phosphorus (%)	0.45	0.45	0.45	0.45	0.45	0.45	0.45
Calcium (%)	0.92	0.92	0.92	0.92	0.92	0.92	0.92
Sodium (%)	0.22	0.22	0.22	0.22	0.22	0.22	0.22
Choline (p.p.m.)	1,600	1,600	1,600	1,600	1,600	1,600	1,600
Digestible lysine (%)	1.07	1.07	1.07	1.07	1.07	1.07	1.07
Digestible TSAA (%)	0.80	0.80	0.80	0.80	0.80	0.80	0.80
Digestible threonine (%)	0.72	0.72	0.72	0.72	0.72	0.72	0.72
Digestible tryptophan (%)	0.23	0.20	0.20	0.20	0.20	0.20	0.20

T1: control diet; T2: raw cowpeas; T3: roasted cowpeas; T4: raw cowpeas with enzymes; T5: raw pigeon peas; T6: roasted pigeon peas; T7: Raw pigeon peas with enzymes. Digestible TSAA, digestible total sulfur amino acids; ME, metabolizable energy.

**TABLE 3:** Chemical composition in Cowpeas (*Vigna unguiculata*) and Pigeon peas (*Cajanus cajan*).

Component	Cowpea	Pigeon pea
Moisture %	12.45	11.28
Crude protein %	22.75	21.07
Crude fat %	1.80	1.41
Crude fiber %	4.73	6.48
Ash %	3.42	3.44

**TABLE 4:** Antinutritional factors in raw and roasted cowpea and pigeon pea.

Treatment	Trypsin Inhibitor TIU/g		Urease Assay		Phytic Acid %		Tannins %	
	Cowpea	Pigeon pea	Cowpea	Pigeon pea	Cowpea	Pigeon pea	Cowpea	Pigeon pea
Raw	3000–10400	7650 (5800–9500)	0.03	2.69	1.2	0.57	0.86	0.7
Roasted	<2000	<2000	−0.245	1.73	0.86	0.34	1.12	0.59

### 2.7 Measurements

#### 2.7.1 Gut health analysis based on digestive tract histopathology

On day 12, two birds per pen were randomly selected (n = 70 birds), individually weighed, and injected with bromodeoxyuridine (BRDU) at a dose of 10 mg/kg (BD Bioscience, Switzerland) as a marker for gut epithelial growth, following the manufacturer’s instructions. Two days later, the same birds received a second BRDU injection before being weighed and euthanized. Tissue samples were collected from each bird, including a 1 cm segment of the duodenum (empty), a 1 cm segment of the midgut (empty), the ileocecal junction with 1 cm of the attached ileum, and a cross-section of the mid-cecal pouch. The luminal contents were gently flushed with Notoxhisto fixative (Scientific Device Laboratory, Des Plaines, IL), and the tissues were preserved in labeled bottles containing 10–20× the tissue volume of Notox. Samples were pooled by treatment. Also, a 1 cm segment of the duodenum was collected from one bird per pen, flushed with Notox, and fixed in Notox solution at a 10–20× volume ratio in a labeled bottle.

The fixed tissues were processed and embedded in paraffin wax. Sections of 5 µm thickness were obtained and stained with hematoxylin and eosin (H&E) or immunostained with anti-BRDU (MBL, Japan) and anti-IgA. The stained sections were examined using an Olympus light microscope (Evident, Japan). H&E-stained slides were analyzed for gut morphology and morphometric measurements, anti-BRDU-stained slides were used to assess cell proliferation, and anti-IgA-stained slides were examined for immune response (Keel et al., 2022; Leiva et al., 2022). For each sample, five randomly selected villi were measured for height and width, five crypts for depth, and the mucosal tissue thickness was assessed at five different locations. Measurements were taken using an Olympus light microscope with a 10× eyepiece lens and a ×10 objective lens, providing a total magnification of ×100 (Marchewka et al., 2021). The villus height, width, crypt depth, crypt depth-to-villus height ratio (CV), and mucosal tissue thickness (TMUC) were compared across dietary treatments. Increased villus height and decreased villus breadth imply a larger absorptive surface area, whereas reduced crypt depth and a lower crypt depth-to-villus height ratio indicate a lesser need for cell proliferation to keep the gut intact. The mean values of five villus heights, widths, crypt depths, and TMUC measurements per sample were used for statistical analyses.

#### 2.7.2 Digesta viscosity assessment

Broilers from all treatment groups were utilized for viscosity measurements. Jejunal and ileal contents from two randomly selected birds per pen were collected (n = 70 birds). The digesta were carefully extracted and transferred onto pre-weighed weigh boats, after which their weights were recorded (Novotný et al., 2023). The collected digesta samples were centrifuged at 2400 r.p.m. for 10 min. Subsequently, 0.5 mL of the supernatant was meticulously withdrawn and placed into a viscometer sample cup. The cup was then affixed to the viscometer, and each sample was allowed a 1-min equilibration period before measurement. Viscosity was assessed using a Brookfield LVDV-1 viscometer (Brookfield Engineering Laboratories, Middleboro, MA, USA) at a controlled temperature of 25°C and a rotational speed of 100 r.p.m.

#### 2.7.3 Performance of *Clostridium perfringens* cultures

Tied-off ileums from two randomly selected broilers per pen were collected and stored in saline at refrigeration temperature for subsequent *Clostridium* culture (Zhao et al., 2022). Ileal digesta samples were transferred into sterile 50 mL tubes, precisely weighed to the nearest 0.1 g, and diluted (w/w) with sterile phosphate-buffered saline to achieve 10× (0.1), 1000× (0.001), and 1,000,00× (0.00001) dilutions. One milliliter of each dilution was dispensed into sterile Petri dishes, followed by 20 mL of molten SPS agar (BD Diagnostic Systems, Sparks, MD) heated to 48°C.

The plates were gently swirled to ensure thorough mixing and allowed to solidify. Once solidified, the plates were placed into BD EZ anaerobe gas pack pouches to establish an anaerobic environment and incubated at 37°C for 18 h. After incubation, all black colonies were enumerated.

#### 2.7.4 Growth performance

Broilers were weighed by pen on days 0, 7, and 14. Feed consumption was assessed by transferring any remaining feed from the feeder back into the corresponding pail, weighing the pail with the feed, and subtracting the weight of the empty pail from the total. Mortality was monitored twice daily, and the weights of deceased birds were recorded to adjust feed conversion calculations. Body weight gain was determined as the difference between successive body weight measurements. The feed-to-gain ratio, adjusted for mortality, was calculated as follows:

((cumulative livability x ((body weight x 1000)/Day of study) x 10)/(cumulative feed to gain corrected for dead bird weight)) (Yerpes et al., 2020).

#### 2.7.5 Apparent ileal digestibility calculation

On day 15, ileal digesta from all remaining birds (n = 135) and excreta from all pens were collected for digestibility analysis. Apparent digestibility was calculated using the following equation:

Apparent ileal digestibility coefficient % = 1 - ((Nutrient in excreta x AIA in excreta)/(Nutrient in diet x AIA in diet)) (Beckman et al., 2024).

### 2.8 Statistical analyses

Statistical analyses were performed using analysis of variance (ANOVA) appropriate for a randomized complete block design (RCBD). The General Linear Models (GLM) procedure in SAS (SAS Institute, 2011) was employed to evaluate treatment effects. When a significant effect was detected (*p < 0.05*), mean comparisons were conducted using Fisher’s protected least significant difference (LSD) method to identify differences among treatments. A significance threshold of *p < 0.05* was applied to all analyses to ensure statistical rigor.

## 3 Results

### 3.1 Growth performance

The effects of dietary treatments on chick growth performance are presented in Table 5. By day 7, body weight gain (BWG) and the cumulative performance index (CPI) of chicks fed raw cowpeas or raw pigeon peas were significantly lower than those of chicks fed the control diet (*p < 0.05*). Neither roasting cowpeas nor supplementing raw cowpeas with enzymes improved chick performance beyond that of the raw cowpea group (*p > 0.05*). BWG and CPI of chicks fed roasted cowpeas were comparable to those fed raw cowpeas (*p > 0.05*) but remained significantly lower than those observed in the control group (*p < 0.05*). Also, no significant differences were found between chicks fed raw cowpeas and those receiving raw cowpeas supplemented with enzymes (*p > 0.05*).

**TABLE 5:** Effects of cowpeas and pigeon peas on 7 and 14 days growth performance of chicks fed diets containing enzymes and raw or roasted beans.

Day	Parameter	T1 (Control)	T2 (Raw Cowpeas)	T3 (Roasted Cowpeas)	T4 (Raw Cowpeas + Enzymes)	T5 (Raw Pigeon Peas)	T6 (Roasted Pigeon Peas)	T7 (Raw Pigeon Peas + Enzymes)	P-value
7	Body Weight (kg)	0.141^a^	0.116^b^	0.124^bc^	0.110^bc^	0.123^bc^	0.131^ac^	0.115^bc^	0.0009
	Body Weight Gain (kg)	0.101^a^	0.077^b^	0.084^b^	0.070^bc^	0.083^bc^	0.090^ab^	0.080^bc^	0.0007
	Feed Intake (g/day/bird)	1.353^a^	1.623^b^	1.534^ab^	1.749^c^	1.683^b^	1.421^a^	1.600^b^	0.0012
	Cumulative Performance Index (CPI)	148.78^a^	101.24^b^	105.29^b^	82.56^c^	92.75^c^	132.37^ab^	90.75^c^	0.0023
	Mortality (%)	0.00^a^	3.33^a^	12.50^b^	10.00^b^	12.50^b^	0.00^a^	12.50^b^	0.0001
14	Body Weight (kg)	0.390^a^	0.307^b^	0.314^b^	0.296^b^	0.319^b^	0.360^a^	0.300^b^	0.0001
	Body Weight Gain (kg)	0.249^a^	0.191^b^	0.190^b^	0.186^b^	0.196^b^	0.229^a^	0.198^b^	0.0001
	Feed Intake (g/day/bird)	1.389^a^	1.621^a^	1.603^a^	1.549^a^	1.745^b^	1.573^a^	1.780^b^	0.0001
	Cumulative Performance Index (CPI)	188.99^a^	132.43^b^	119.08^b^	119.22^b^	115.03^b^	145.18^b^	125.03^b^	0.0001
	Mort%	0.00^a^	3.33^a^	12.50^b^	10.00^b^	12.40^b^	0.00^a^	12.45^b^	0.0001

Different superscripts (a–c) within a row indicate statistically significant differences among treatments (*p < 0.05*).

Key: BWT, body weight; BWG, body weight gain; Fgdb = feed gain per day per bird; CPI, cumulative performance index; Mort% = mortality percentage. T1: control diet; T2: raw cowpeas; T3: roasted cowpeas; T4: raw cowpeas with enzymes; T5: raw pigeon peas; T6: roasted pigeon peas; T7: raw pigeon peas with enzymes.

Chicks fed roasted pigeon peas exhibited BWG, feed consumption (FC), and CPI similar to those of the control group (*p > 0.05*). However, mortality was significantly higher among chicks consuming raw beans, roasted cowpeas, roasted cowpeas with enzyme supplementation, raw pigeon peas, and raw pigeon peas with enzymes compared to those fed the control diet or roasted pigeon peas (*p < 0.05*). By day 14, BWG and CPI were significantly lower in chicks fed raw cowpeas, roasted cowpeas, roasted cowpeas with enzymes, raw pigeon peas, or raw pigeon peas with enzymes compared to those receiving the control diet (*p < 0.05*).

### 3.2 Digesta viscosity

**FIGURE 1:**
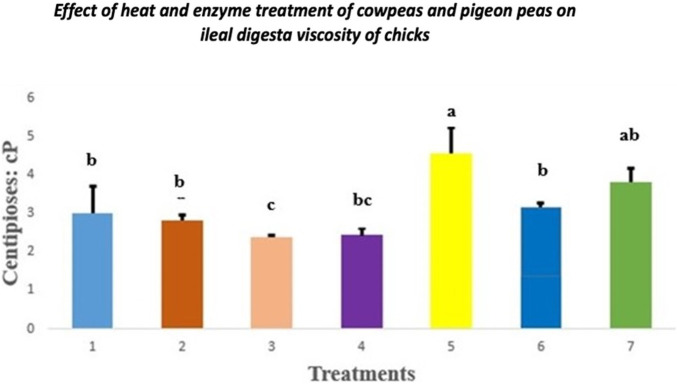
Effect of heat and enzyme treatment of cowpeas and pigeon peas on ileal digesta viscosity in chicks. Different letters **(a–c)** among bars indicate statistically significant differences among treatments (*p < 0.05*). T1: Control diet; T2: Raw cowpeas; T3: Roasted cowpeas; T4: Raw cowpeas with enzymes; T5: Raw pigeon peas; T6: Roasted pigeon peas; T7: Raw pigeon peas with enzymes.

The effect of heat and enzyme treatment of cowpeas and pigeon peas on digesta viscosity in chicks is presented in Figure 1. Chicks fed raw cowpeas showed significantly lower jejunal digesta viscosity than those fed raw pigeon peas (*p < 0.05*). However, no significant differences in digesta viscosity were observed among chicks fed other dietary treatments compared to the control group (*p > 0.05*).

### 3.3 *Clostridium* growth

**FIGURE 2:**
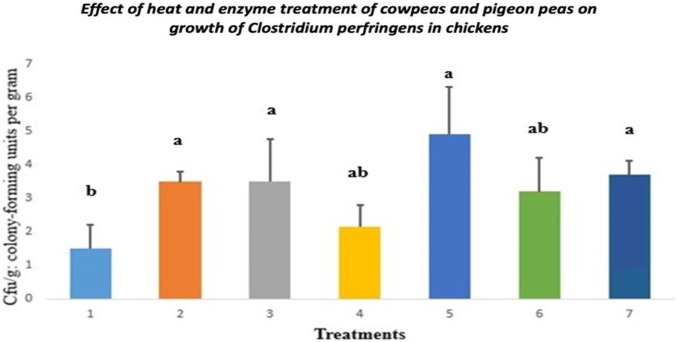
Clostridial growth in chicks fed cowpeas and pigeon peas. Different letters (a–c) among bars indicate statistically significant differences among treatments (*p < 0.05*).T1: Control diet; T2: Raw cowpeas; T3: Roasted cowpeas; T4: Raw cowpeas with enzymes; T5: Raw pigeon peas; T6: Roasted pigeon peas; T7: Raw pigeon peas with enzymes. Cfu/g = Colony-forming units per Gram.

The impact of heat and enzyme treatment of cowpeas and pigeon peas on the growth of *Clostridium perfringens* in chickens is illustrated in Figure 2. Chicks fed raw cowpeas and those fed raw cowpeas supplemented with enzymes exhibited similar levels of *Clostridium* growth to those fed the control diet (*p > 0.05*). The *Clostridium* colonies formed in chicks fed roasted cowpeas were not significantly different from those observed in chicks fed raw pigeon peas (*p > 0.05*). Chicks fed roasted cowpeas, raw pigeon peas, and raw pigeon peas with enzyme supplementation had significantly more *Clostridium* colonies than those fed the control diet (*p < 0.05*).

### 3.4 Duodenal morphology

The effects of heat and enzyme treatments of cowpeas and pigeon peas on villus length and width, crypt depth, and mucosal tissue thickness in broilers were evaluated. The results revealed no significant differences in mucosal tissue thickness, villus length, or villus width among birds fed the different dietary treatments (*p > 0.05*). However, chicks fed raw cowpeas, raw cowpeas supplemented with enzymes, and raw pigeon peas (both with and without enzymes) exhibited significantly reduced duodenal crypt depths compared to those fed the control diet (*p < 0.05*). In contrast, chickens fed roasted cowpeas and roasted pigeon peas showed no significant differences in duodenal crypt depths compared to those on the control diet (*p > 0.05*).

### 3.5 Immune function

**FIGURE 3:**
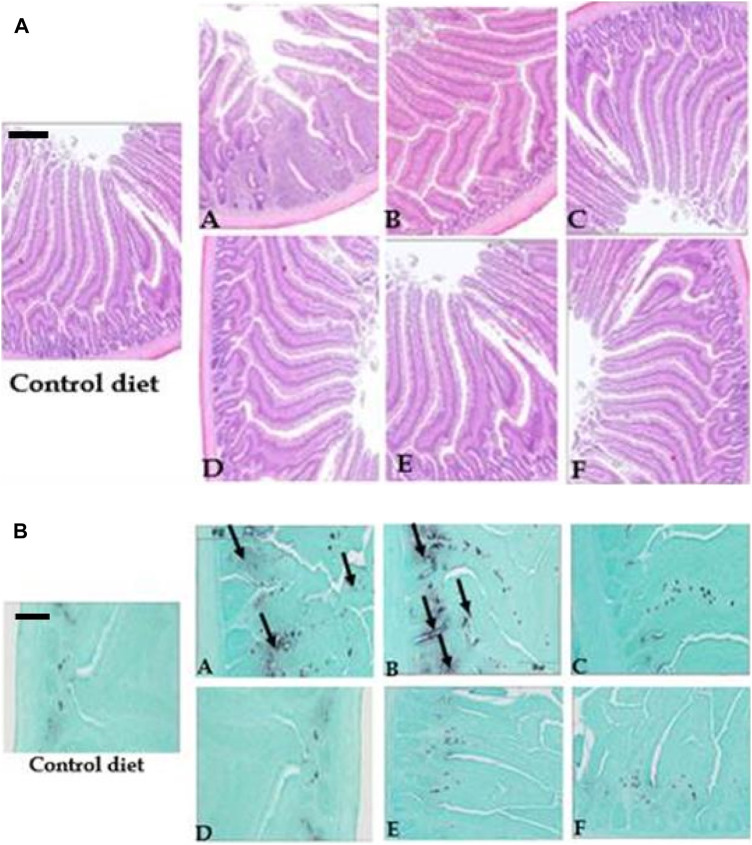
**(A)** Morphology of the duodenum in chicks fed corn-soy, raw cowpeas, roasted cowpeas, raw cowpeas with enzymes, raw pigeon peas, and roasted pigeon peas (H&E staining, ×40 magnification). Control diet–Long, thin villi; no inflammation. (A) Raw cowpeas–Short, thin villi; no inflammation. (B) Roasted cowpeas–Short, thicker villi; substantial inflammation. (C) Raw cowpeas with enzymes–Long, thicker villi; no inflammation. (D) Raw pigeon peas–Long, thicker villi; no inflammation. (E) Roasted pigeon peas–Long, thicker villi; no inflammation. (F) Raw pigeon peas with enzymes–Long, thicker villi; no inflammation. Scale bar = 400 µm. **(B)** Morphology of duodenum from chicks fed corn-soy, raw cowpeas, roasted cowpeas, raw cowpeas with enzymes, raw pigeon peas, and roasted pigeon peas (H&E, 40x); IgA = Immunoglobulin. Control diet – No IgA (Black). A. Raw cowpeas diet – Significant IgA presence (Black). B. Roasted cowpeas – More IgA (Black) than corn-soy but less than raw cowpeas. C. Raw cowpeas with enzymes – Substantial inflammation. D. Raw pigeon peas – No IgA (Black). E. Roasted pigeon peas – No IgA (Black). F. Raw pigeon peas with enzymes – No IgA (Black). Scale bar: 400 µm.

Raw cowpeas, both with and without enzyme supplementation, elicited an immune response in the duodenum (Figure 3a), a response that was absent in the control diet and significantly diminished by roasting (Figure 3b). In contrast, pigeon peas (with or without enzymes) exhibited a lower immunogenicity than cowpeas. The most pronounced immune response was observed in chicks fed raw cowpeas. Also, chicks fed raw pigeon peas demonstrated greater cellular proliferation in the villus core compared to those fed roasted cowpeas, as indicated by increased immunoglobulin A (IgA) staining in the duodenum of chicks fed raw pigeon peas relative to those fed roasted pigeon peas (Figure 3b).

### 3.6 Ileal apparent digestibility

Table 6 presents the results of dietary treatments on dry matter (DM) digestibility.

**TABLE 6:** Apparent ileal dry matter and nitrogen digestibility in the diets containing enzyme and raw or roasted beans.

Treatment	Apparent dry matter digestibility	Apparent nitrogen digestibility
T1 (Control)	0.73^a^	0.21^a^
T2 (Raw Cowpeas)	0.72^a^	0.34^b^
T3 (Roasted Cowpeas)	0.91^b^	0.73^c^
T4 (Raw Cowpeas + Enzymes)	0.90^b^	0.73^c^
T5 (Raw Pigeon Peas)	0.84^c^	0.63^d^
T6 (Roasted Pigeon Peas)	0.84^c^	0.71^e^
T7 (Raw Pigeon Peas + Enzymes)	0.83^c^	0.65^d^
S.E.M	0.013	0.018
p-value	0.002	0.003

Different superscripts within a column (a–e) indicate statistically significant differences within each parameter (*p < 0.05*).

T1: control diet; T2: raw cowpeas; T3: roasted cowpeas; T4: raw cowpeas with enzymes; T5: raw pigeon peas; T6: roasted pigeon peas; T7: Raw pigeon peas with enzymes. S.E.M, standard error of the mean.

### 3.7 Apparent ileal digestibility

Chicks fed heat-treated or raw cowpeas supplemented with enzymes had higher apparent ileal dry matter digestibility than those fed raw cowpeas or the control diet (*p < 0.05*). Heat treatment of cowpeas enhanced apparent digestibility compared to raw cowpeas (*p < 0.05*). In contrast, no significant differences were observed in chicks fed raw pigeon peas (with or without enzymes) or roasted pigeon peas (*p > 0.05*). Chicks fed raw cowpeas showed higher ileal nitrogen digestibility than those fed a control diet (*p < 0.05*). Chicks fed raw cowpeas and raw cowpeas supplemented with enzymes exhibited similar apparent ileal nitrogen digestibility (*p > 0.05*). Roasted pigeon peas improved nitrogen digestibility compared to raw pigeon peas (*p < 0.05*). Raw cowpeas showed lower ileal nitrogen digestibility than raw pigeon peas (*p < 0.05*).

## 4 Discussion

The effects of roasted cowpea and pigeon pea-based diets, with or without the addition of a supplemental enzyme mix, on gut health and broiler production performance were evaluated. Body weight gain (BWG) and cumulative performance index (CPI) of chicks fed 400 g/kg raw cowpeas and pigeon peas were lower than those of chicks fed the control diet. Similarly, Uzcátegui-Varela et al. (2020) found that feeding 20% of cowpeas caused lower BWG than the control. Amaefule et al. (2011) discovered that chicks fed 30% or 40% raw pigeon peas had lower BWG, protein efficiency ratio, and feed conversion ratio than those fed a control diet of maize and soy. Moreover, Hervé et al. (2020) found that broiler growth performance decreased as the amount of raw cowpea in the diet increased.

Also, Muamer et al. (2012) observed that cowpea inclusion at 10% reduced body weight in chicks at 2 weeks of age compared to controls. Anjos et al. (2012) and Abdelgani et al. (2013) reported that chicks fed 20% and 15% raw cowpea had similar BWG to those fed the control diet. The reduced BWG in chicks fed raw cowpeas may be attributed to the high concentration of trypsin inhibitors, which negatively affects chicken growth and gut health (Tshorhote et al., 2003; Belal et al., 2011; Kuenz et al., 2022). Heat treatment, however, reduced the concentration of trypsin inhibitors to below 2000 TIU/g.

Cowpeas and pigeon peas are rich in ANFs such as trypsin inhibitors, phytic acid, tannins and non-starch polysaccharides (NSP), which exhibit anti-nutritive properties that negatively impact chicken performance (Leeson and Summers, 2001; Nguyen et al., 2022). These compounds are known to reduce nutrient digestibility, inhibit enzymatic activity, and damage the gut lining. Bedford (2000) and Ravindran and Abdollahi, (2021) explained that highly digestible diets are absorbed efficiently before creating an environment conducive to bacterial growth. In contrast, poorly digestible diets allow nutrients to escape digestion and absorption, reaching the mid and lower small intestine, where they serve as substrates that promote bacterial growth. So, the microflora consumes a portion of the diet’s energy and protein, leaving less for the host (Bedford and Apajalahti, 2022).

Using enzymes to enhance the growth performance of chickens is well-established (Bedford, 2000; Sundu et al., 2006; Mingbin et al., 2009; Bedford, 2022). Adding a combination of xylanase, protease, and phytase enzymes to raw cowpea diets did not mitigate the negative effects of raw cowpeas, likely because the enzyme mixture did not compensate for the anti-nutritional factors present. In this study, trypsin inhibitor levels in raw cowpeas exceeded 4,000 TIU/g and were reduced by more than 50% through roasting. However, performance in chicks fed roasted cowpeas remained below control levels, likely due to residual ANFs especially tannins, which were initially higher in cowpeas (0.65%) compared to pigeon peas (0.18%). Phytic acid, which binds essential minerals and inhibits nutrient absorption, was moderately reduced by roasting and further hydrolyzed by added phytase. This combination improved apparent ileal digestibility of nitrogen and dry matter, particularly in cowpea diets. However, these gains in digestibility did not always correlate with performance, underscoring the importance of gut health and immune modulation. NSPs, responsible for increased digesta viscosity and slower feed passage, were partially broken down by enzyme supplementation. While enzyme-treated diets showed improved viscosity profiles and nutrient digestibility, especially in raw cowpeas, these biochemical improvements did not consistently restore growth performance. This may be due to lingering mucosal irritation or immune activation, as evidenced by increased duodenal crypt depth reduction and IgA staining in birds fed raw cowpeas with or without enzymes. However, Hervé et al. (2020) concluded that supplementing raw cowpeas with enzymes did not improve broiler growth performance, while Belal et al. (2011) observed a positive effect of adding enzymes to raw cowpeas, with BWG being higher than that of birds fed the control diet.

Amaefule et al. (2011) noted that a diet containing 30% raw pigeon peas required supplementation with methionine, while a 40% inclusion of raw pigeon peas necessitated supplementation with both lysine and methionine to enhance chick growth. These findings suggest that pigeon peas are deficient in both methionine and lysine. Also, methionine supplementation may provide the sulfur required to detoxify the anti-nutritional factors (ANFs) found in raw pigeon pea diets. However, enzyme supplementation did not improve growth performance in these birds.

Earlier research by David et al. (2024) found that chicks fed roasted pigeon peas outperformed those fed raw pigeon peas. Chicks fed 40% roasted pigeon peas had similar BWG, feed gain (FG), cumulative performance index (CPI), and mortality rates as chicks fed a control diet. Ani and Okeke (2011) discovered that including 33% roasted pigeon peas in chick diets reduced feed intake, weight gain, and feed utilization efficiency; however, including 27% roasted pigeon peas in broiler finisher diets had no negative effect on growth performance. These results suggest that heat treatment is effective in reducing anti-nutritional factors in pigeon peas (Table 4).

Maintaining gut health is crucial for the welfare and productivity of chickens, particularly in the absence of antibiotic supplementation (Abd El-Hack et al., 2022). Raw pigeon peas had a higher viscosity than the other legumes studied. Digesta viscosity is positively correlated with feed passage rate through the gastrointestinal tract (Tejeda and Kim, 2021), but no significant differences in digesta viscosity were found between dietary treatments.

Svihus (2011) suggested that poor growth performance may be partially attributed to a reduced feed passage rate. Enzyme supplementation in poultry diets, especially those high in non-starch polysaccharides (NSP), can reduce dietary and digesta viscosity, increasing nutrient utilization (Khattak et al., 2006). In this study, adding enzymes to the raw cowpea diet increased digesta viscosity compared to the control diet. Also, roasting raw pigeon peas reduced digesta viscosity compared to raw pigeon peas, indicating that processing or enzyme supplementation can mitigate the negative effects of raw legumes.

Gut microflora plays a critical role in modulating the immune status of birds by influencing the intestinal wall, with diet being a key determinant of microbial composition (Choct, 2009; Wickramasuriya et al., 2022). In this study, chicks fed roasted cowpeas and raw pigeon peas had higher *C. perfringens* colony counts than those fed a control diet. This variation may be attributed to differences in tannin concentrations between the two legumes. Price et al. (1980) reported tannin levels ranging from 0% to 0.7% in cowpeas and 0%–0.2% in pigeon peas, suggesting that roasting may not have been adequate to eradicate tannins in cowpeas. Tosi et al. (2013) proposed that tannin extracts from chestnuts could help control necrotic enteritis and inhibit the proliferation of *C. perfringens* in broiler chickens.

Dietary treatments had no significant effects on mucosal tissue thickness, villus length, or villus width. However, the poor growth performance observed in chicks fed raw cowpeas, raw cowpeas supplemented with enzymes, and raw pigeon peas was consistent with their reduced crypt depth compared to those fed the control diet. Histological staining of duodenal tissue for IgA indicated a potential immunological benefit associated with roasting cowpeas.

Increased digesta viscosity and changes in gut morphology likely contribute to significant alterations in broiler performance, particularly in young birds (Yasar, 2003). In this study, roasting pigeon peas improved apparent ileal nitrogen digestibility, aligning with the observed improvements in growth performance. In contrast, the increased apparent ileal nitrogen digestibility found in all cowpea treatments did not translate into improved growth performance, indicating that roasting alone was insufficient to entirely ameliorate the deleterious impacts of anti-nutritional elements in cowpeas. However, enzyme treatment enhanced cowpeas' apparent ileal nitrogen and dry matter digestibility. Also, Iyayi (2013) found that supplementing a diet containing 30% roasted cowpeas (heated to 50°C) with 500 units of phytase increased the ileal digestibility of phosphorus, crude protein, and amino acids. Moreover, phytase supplementation enhanced feed intake and body weight gain, findings that contrast with those of the present study.

This study has important implications for the poultry industry and sustainable livestock nutrition, particularly in sub-Saharan Africa. The demonstration that roasted pigeon peas can successfully replace up to 40% of conventional protein sources without compromising growth or gut health offers a cost-effective and locally viable alternative to soybean meal. This is particularly relevant as soybean meal becomes increasingly expensive and environmentally burdensome.

In regions like Mozambique, where pigeon peas and cowpeas are already cultivated often by women and smallholder farmers this finding promotes greater feed self-reliance, food security, and gender-inclusive rural development. Roasting, a simple and accessible processing method, adds value to local crops without requiring industrial infrastructure, thus bridging the gap between scientific innovation and field-level feasibility.

From an environmental standpoint, replacing imported feed ingredients with locally sourced legumes can significantly reduce the carbon footprint of poultry production, support agroecological principles, and foster resilient food systems. These insights align with global goals for climate-smart agriculture and point toward the strategic importance of feed diversification in achieving both economic and ecological sustainability in poultry farming.

Despite its valuable findings, this study has several limitations. First, the experimental duration was restricted to the starter phase (0–14 days post-hatch), limiting insights into longer-term effects on carcass traits, immune resilience, and economic returns. Second, only male Ross 708 broilers were used, which may not reflect the responses of indigenous or dual-purpose breeds more common in rural or smallholder systems.

Third, although roasting and enzyme supplementation were evaluated, other processing techniques such as fermentation, soaking, or germination were not tested but may offer additional benefits in reducing ANFs. The study also relied primarily on histological measures of gut health without incorporating microbial or systemic immune analyses, which would provide deeper mechanistic insights.

## 5 Conclusion

This study aimed to evaluate the effects of roasting and enzyme supplementation on the nutritional utilization, gut health, and growth performance of broiler chickens fed diets containing cowpeas (*V. unguiculata*) and pigeon peas (*C. cajan*) at a 400 g/kg inclusion rate, replacing soybean meal. The findings confirm that raw cowpeas and pigeon peas negatively affect broiler performance and gut morphology, primarily due to the presence of anti-nutritional factors such as trypsin inhibitors, phytic acid, and tannins. Roasting effectively reduced these anti-nutritional compounds, especially in pigeon peas, resulting in improved performance metrics (BWG, CPI), gut histology, and nitrogen digestibility comparable to the conventional maize–soybean control diet. In contrast, roasting of cowpeas and the addition of exogenous enzymes, while improving digestibility, did not fully restore growth performance or eliminate signs of immune activation, indicating that cowpeas may require more intensive or combined processing strategies to be viable in poultry diets. Importantly, the results demonstrate that roasted pigeon peas can be used to partially replace soybean and maize meal up to 40% of the diet without compromising broiler growth or gut health, making it a promising alternative protein source in regions where conventional feed ingredients are scarce or costly. This has strong practical implications for poultry feed formulation, particularly in smallholder systems in sub-Saharan Africa, where access to imported feed components is limited. The study underscores the need for legume-specific processing strategies and supports the integration of locally available feed resources into broiler diets as a cost-effective and sustainable solution. Further research is warranted to evaluate these findings over longer production cycles, with additional legume processing techniques and across diverse broiler genotypes.

## Data Availability

The datasets presented in this study can be found in online repositories. The names of the repository/repositories and accession number(s) can be found in the article/supplementary material.
